# The protection motivation theory as an explanatory model for intention to use alcohol protective behavioral strategies related to the manner of drinking among young adults

**DOI:** 10.1093/alcalc/agae059

**Published:** 2024-08-30

**Authors:** Bella M González-Ponce, José Carmona-Márquez, Angelina Pilatti, Carmen Díaz-Batanero, Fermín Fernández-Calderón

**Affiliations:** Department of Clinical and Experimental Psychology, University of Huelva, Huelva, Spain; Research Center on Natural Resources, Health and the Environment, University of Huelva, Huelva, Spain; Department of Clinical and Experimental Psychology, University of Huelva, Huelva, Spain; Research Center on Natural Resources, Health and the Environment, University of Huelva, Huelva, Spain; Faculty of Psychology, National University of Córdoba, Córdoba, Argentina; Institute of Psychological Research, IIPsi-CONICET-UNC, Universidad Nacional de Córdoba, Córdoba, Argentina; Department of Clinical and Experimental Psychology, University of Huelva, Huelva, Spain; Research Center on Natural Resources, Health and the Environment, University of Huelva, Huelva, Spain; Department of Clinical and Experimental Psychology, University of Huelva, Huelva, Spain; Research Center on Natural Resources, Health and the Environment, University of Huelva, Huelva, Spain

**Keywords:** alcohol, protective behavioral strategies, manner of drinking, protection motivation theory, intention, young adults

## Abstract

**Aims:**

This study aimed to prospectively examine the explanatory value of the protection motivation theory (PMT) for the intention to use manner of drinking protective behavioral strategies (MD PBS) and to explore its invariance across genders.

**Method:**

A targeted sampling procedure was used to recruit 339 young adults in the community (*M*_age_ = 21.1; SD = 2.21; female = 50.7%) who completed baseline and 2-month follow-up measures of the PMT constructs and intentions to use each of the five MD PBS.

**Results:**

Regression analyses revealed that the coping appraisal components (response efficacy and self-efficacy) had greater explanatory power for the intention to use MD PBS than the threat appraisal components (perceived vulnerability and perceived severity). Perceived vulnerability to alcohol consequences was not prospectively associated with any specific behavioral intention or with the total MD PBS score. In contrast, perceived severity was prospectively associated with the intention to use three out of five PBS and the total MD score. Regression coefficients revealed gender invariance for all six models.

**Conclusions:**

Our findings suggest that interventions aimed at encouraging young adults to use alcohol MD PBS would be most effective if they included components that enhance self-efficacy in using these strategies and emphasize their perceived usefulness in reducing alcohol-related consequences.

## Teaser

**Short Summary:** This study has shown that the coping appraisal components of the protection motivation theory better explain the motivation to use alcohol protective behavioral strategies related to the manner of drinking among young adults who use alcohol than the threat appraisal components. Additionally, the results of this model were invariant across genders.

## Introduction

Alcohol is the most widely consumed substance worldwide, with its use especially prevalent among young adults (World Health Organization [WHO] 2022). Alcohol use—particularly heavy drinking—has been associated with numerous negative consequences, including physical illness, mental health problems, risky sexual behavior, and unsafe driving (WHO 2022). Additionally, excessive consumption also increases the likelihood of developing an alcohol use disorder (Prince et al. 2019). Previous research has shown that young adults use protective behavioral strategies (PBS; Martens et al. 2005), defined as cognitive–behavioral strategies to minimize potential alcohol-related consequences. Three subtypes of alcohol PBS have been typically identified: serious harm reduction (SHR) strategies (e.g. eating before or during drinking), stopping/limiting drinking (SLD) strategies (e.g. alternating between alcoholic and nonalcoholic drinks), and manner of drinking (MD) strategies. These latter strategies include avoiding drinking games or mixing different types of alcohol; drinking slowly rather than gulping or chugging; avoiding trying to keep up or outdrink others; and avoiding pregaming. Previous literature consistently indicates that, when considered together, PBS are associated with decreased alcohol consumption and its consequences. However, variability exists among the different types of PBS (Peterson et al. 2021; Cox et al. 2024). Specifically, SLD PBS have shown the weakest relationships with both alcohol use and its consequences (Cox et al. 2024). In contrast, SHR PBS have proven effective in mitigating negative consequences (Richards et al. 2018; Peterson et al. 2021), while MD PBS have been consistently linked to heavy alcohol consumption and alcohol-related negative consequences (Peterson et al. 2021; Cox et al. 2024). Given this context, our study focuses specifically on MD strategies, as they primarily target alcohol consumption patterns while also impacting negative consequences.

Given the demonstrated importance of PBS in reducing alcohol-related negative consequences, numerous studies in recent years have analyzed the explanatory factors of their use (for a review, see González-Ponce et al. 2022b). These factors include the perceived efficacy of PBS in reducing alcohol-related negative consequences (González-Ponce et al. 2022a) along with descriptive social norms concerning PBS use within peer groups (Fernández-Calderón et al. 2022), among others. Although identifying these factors is valuable for designing interventions to promote PBS use, theory-based explanatory models have shown greater utility in explaining the development, maintenance, and change in patterns of health-related behaviors (Conner and Norman 2015). In addition, health promotion interventions based on theoretical models are more effective than those based on isolated constructs (e.g. DiClemente et al. 2013). Thus, testing a model that theoretically integrates different constructs shown to explain protective behaviors against alcohol consumption (i.e. PBS use) may be useful for increasing the effectiveness of theory-based interventions to encourage PBS use and reduce heavy drinking. However, to our knowledge, the literature on theoretical models for explaining MD PBS is scarce. In particular, Scaglione et al. (2015) demonstrated the utility of a dual-process decision-making model using a rational pathway (i.e. intentions) and reactive pathway (i.e. willingness) for predicting PBS use among college students. Specifically, after controlling for age, typical drinking, and previous PBS use, expectancies and attitudes were positively associated with using PBS through the rational pathway (i.e. intentions). Additionally, self-concept was associated with increased PBS use through willingness, while injunctive norms were positively associated with PBS use through intentions and willingness.

The protection motivation theory (PMT; Maddux and Rogers 1983; Rogers 1983) is one of the most widely used theoretical models in the field of health psychology, having shown a high predictive power for intention to engage in various health-protective behaviors, including smoking cessation (Maddux and Rogers 1983) and condom use (Chambers et al. 2018). This theory was developed to explain the motivation (operationalized as an intention) to engage in health-protective behaviors in the face of a potential health threat. In particular, as posed by the revised theory formulated by Maddux and Rogers (1983), the intention to adopt a health-protective behavior is expected to depend on two cognitive processes triggered when a health threat appears: threat appraisal and coping appraisal. Threat appraisal is determined by the likelihood of experiencing the threat (perceived vulnerability) and the perceived severity of the health threat, while coping appraisal is composed of beliefs about whether a protective behavior (e.g. using PBS) will be effective in reducing the threat (response efficacy) and the belief in one’s ability to execute the coping response successfully (self-efficacy). According to the postulates of PMT, the intention to engage in a protective behavior (e.g. MD PBS) in the face of a threat (e.g. experiencing negative alcohol-related consequences) will be greater when the individual perceives the threat (e.g. alcohol-related consequences) as serious and probable (threat appraisal), possesses the skills to execute such behavior (e.g. skills to enact MD PBS), and believes that the protective behavior will be effective in reducing the threat (e.g. MD PBS will be effective in reducing alcohol-related consequences; coping appraisal). Given that this model was designed to explain motivations to enact health-related protective behaviors against health threats, we consider it appropriate for explaining the mechanisms through which young adults protect themselves against the threat of potential alcohol-related negative consequences.

While numerous studies (e.g. Floyd et al. 2000) have yielded support for this model in the context of various health-promoting behaviors, several systematic reviews and meta-analyses (e.g. Ruiter et al. 2014) have shown that the coping appraisal component (perceived self-efficacy and response efficacy) has greater explanatory power than threat appraisal. In addition, intention has typically emerged as the strongest predictor of behavior (Ajzen 1991), considered the last step of the motivational phase of decision-making (Conner and Norman 2015). In the field of alcohol use, the coping appraisal component is relevant in explaining PBS use (e.g. González-Ponce et al. 2022a). Additionally, intention to use PBS among university students has been associated with PBS use (Fairlie et al. 2021). Applied to MD PBS, this approach would make it possible to identify the key target constructs (coping or threat) and select appropriate intervention components to increase motivation among young adults to use these strategies. Understanding the explanatory power of PMT and its various constructs for the intention to use alcohol MD PBS could inform the development of interventions based on this theory. These interventions could potentially be effective in reducing alcohol use and related harms (e.g. Hagger and Weed 2019).

Previous studies have shown that women experience more alcohol-related negative consequences than men (Patrick et al. 2020). Moreover, women’s affective response to threatening stimuli is more negative (Wen et al. 2022), leading them to take more protective measures against the same threats than men (Guo et al. 2015). Consistent with these observations, previous research has demonstrated that women use the three types of PBS more frequently than men (e.g. Tabernero et al. 2019; Schwebel et al. 2022). Regarding MD PBS, women are more likely to use these protective behaviors than men (e.g. LaBrie et al. 2011; Miller et al. 2019). Specifically, it has been observed that, compared to men, women report higher use of three (avoid drinking games, avoid mixing different types of alcohol, and avoid trying to “keep up” with or outdrink others) of the five MD PBS (e.g. Schwebel et al. 2022). The use of these strategies is associated with greater reductions in alcohol-related consequences for women than for men (Clarke et al. 2016; Schwebel et al. 2022). The explanatory factors of MD PBS use (e.g. perceived vulnerability and perceived severity of the threat) may impact males and females differently and produce higher or lower use of PBS. Thus, examining whether there are gender differences in the explanatory power of the PMT for MD PBS use could be informative for designing targeted gender-based interventions for promoting the use of these strategies.

Considering the above, we aimed to (i) prospectively examine the explanatory value of the PMT for the intention to use alcohol PBS related to the MD strategies and (ii) examine whether this explanatory model varies across genders. Based on the previous literature, it is hypothesized that the coping appraisal components will show greater explanatory power than the threat appraisal components. However, given the lack of research that has examined gender differences in the explanatory value of the PMT for PBS use, no hypothesis is proposed in this regard.

## Materials and Methods

### Participants and procedure

Participants were young adults aged 18–25 years (*M* = 21.1, *SD* = 2.2; 50.7% female) who reported using alcohol on at least two occasions in the past month. They were recruited through targeted sampling ( Watters and Biernacki 1989) from several predetermined community locations in the province of Huelva, such as parks and bars. A psychologist with research experience was responsible for recruitment and administering the questionnaires. Posters were also used for recruitment, and following the targeted sampling protocol (Watters and Biernacki 1989), snowball sampling was employed.

Out of the total sample (*n* = 360), 48.3% (*n* = 174) were recruited directly by the fieldworker, 43.1% (*n* = 155) were referred by other participants, and 8.6% (*n* = 31) contacted the researcher after seeing a poster. Participants provided informed consent before participating and completed the questionnaires in paper-and-pencil format in designated rooms at the University of Huelva. Each participant was compensated with a 15-euro Amazon voucher.

From the baseline sample (*n* = 360), 94.2% (*n* = 339) completed a 2-month follow-up. Similar to the baseline measure, participants received an Amazon voucher for 15 euros. Further details of this procedure can be found in Fernández-Calderón et al. (2021).

Among those who participated in the follow-up, 96.2% reported being born in Spain, and 59.0% were studying at a university. The main sources of income reported were a family allowance (51.6%) or a paid job (25.1%), and 77.6% lived with their parents. At baseline, the mean number of drinking and binge drinking days in the past 2 months was 15.8 (*SD* = 11.5) and 5.7 (*SD* = 7.2), respectively. Participants reported consuming between 0 and 63 alcoholic drinks per week, with a mean of 13.84 drinks (*SD* = 11.81) during a typical week in the past month. This corresponds to a mean of 20.61 (*SD* = 18.20) Standard drinking units (SDU).

No statistically significant differences were found between those who participated in the follow-up survey (*n* = 339) and those who did not (*n* = 21) in terms of gender, age, and mean days of drunkenness in the past 2 months (all *p* > 0.05). However, significant differences were found regarding the mean days of alcohol use in the past 2 months (Mann–Whitney *U* = 2628; *z* = −2.016, *P* = 0.044). It was found that those who participated in the follow-up reported more days of consumption (*M* = 15.79; *SD* = 11.54) than those who did not (*M* = 10.62; *SD* = 7.19).

The present study was approved by the Regional Bioethics Research Committee of Andalusia (Consejería de Sanidad, Junta de Andalucía, Spain).

### Measures

The following measures were included in the final questionnaire:

***Alcohol use measures** (baseline):* participants reported the number of days they consumed alcohol and the number of days they were drunk in the last 2 months. To assess the amount of alcohol consumption, participants completed a modified version of the Daily Drinking Questionnaire ( Collins et al. 1985). Following the guidelines of the Spanish Observatory of Drugs and Addictions (Observatorio Español de las Drogas y las Adicciones 2022), participants were provided with information and images of six types of alcoholic beverages. They were asked to “think about a typical week in the last 30 days and indicate how many different types of drinks you consumed on each day of that week.” The number of drinks consumed daily was summed to calculate the total number of alcoholic drinks taken in a typical week in the past month. The quantities for each type of beverage were then converted into SDU.

***Perceived vulnerability to alcohol consequences** (baseline):* following previous studies (e.g. Vera et al. 2022), two items were used to measure perceived vulnerability to experiencing alcohol-related consequences. Participants were asked to indicate how likely they were to experience negative health consequences from drinking alcohol (Item 1) and getting drunk (Item 2) in a five-point response format (1- very unlikely to 5- very likely). Responses were summed to obtain a total score of perceived vulnerability. The Spearman–Brown reliability coefficient for perceived vulnerability was 0.82.

***Perceived severity of alcohol consequences** (baseline):* perceived severity was assessed in a similar way to perceived vulnerability. Thus, following previous research (e.g. Vera et al. 2022), two items were used to ask participants how risky it is to consume alcohol and get drunk (ordinal response format ranging from 1- very unlikely to 5- very likely). The total score was obtained by summing the responses to the two items. The Spearman–Brown reliability coefficient for perceived severity was 0.73.

***Perceived Efficacy of MD PBS to reduce alcohol-related negative consequences** (baseline)*: similar to previous studies (e.g. Ray et al. 2009), we used a modified version of the MD subscale of the PBSS (Treloar et al. 2015) in its Spanish version (Sánchez-García et al. 2020) to measure perceived efficacy of the five PBS. Participants were asked: “Please indicate how effective each of the following behaviors are in reducing alcohol-related negative consequences*.”* We used the same response options as Ray et al. (2009): 1- not at all effective, 2- somewhat effective, 3- moderately effective, and 4-extremely effective. Cronbach's alpha of the perceived efficacy MD scale was 0.77.

***Perceived self-efficacy to engage in MD PBS** (baseline):* based on the recommendations by Ajzen (2006) and previous research in the field of PBS use (Davis and Rosenberg 2016), three items were created to evaluate perceived self-efficacy to use each of the five MD PBS. For example, for the strategy “Avoid mixing different types of alcohol*”*, participants were asked about the extent to which they agree (from 1 = totally disagree to 7 = totally agree) with the following statements: “If I wanted to, I could avoid mixing different types of alcohol”, “If I choose to avoid mixing different types of alcohol when I drink, I can do so*,*” and “If I wanted to, I could easily avoid mixing different types of alcohol*.”* The Cronbach alpha values were as follows: self-efficacy to avoid drinking games =0.89, avoiding mixing =0.79, drinking slowly =0.91, avoiding keeping up =0.87, avoiding pregaming =0.90, and the total score on the MD self-efficacy scale =0.93.

***Intention to use MD PBS** (at follow-up):* as in the case of self-efficacy measurements, we considered Ajzen's recommendations (2006) and previous research (Davis and Rosenberg 2016) to assess the intention to use each MD PBS. For example, for the behavior “Avoid mixing different types of alcohol”, participants were asked to indicate their degree of agreement (from 1 = totally disagree to 7 = totally agree) with the following statements: *“*In the next two months, I am likely to avoid mixing different types of alcohol”*,* “In the next two months, when I drink, I intend to avoid mixing different types of alcohol” and “In the next two months, I will avoid mixing different types of alcohol”*.* Ordinal Cronbach's alpha values were as follows: the intention to avoid drinking games = 0.98, avoiding mixing = 0.93, drinking slowly = 0.96, avoiding keeping up = 0.94, avoiding pregaming =0.96, and total score on intention to use MD PBS scale =0.95.

### Data analysis

Bivariate analysis (Pearson’s correlation) was performed to examine the relationships between the study variables. To mitigate the risk of increased Type I error due to multiple comparisons, we applied the Adjusted Bonferroni procedure (Dunn 1961). This adjustment set the significance level at α_PT_ = 0.05/17 = 0.003.

To address the research questions, we used SPSS 26 (IBM 2019) to conduct six multiple linear regression models, one for each of the five behavior intentions and one for the total score on the MD intention scale as the outcome variables. These models allowed us to estimate the potential associations between PMT variables (perceived vulnerability, perceived severity, response efficacy, and self-efficacy) at baseline and the intentions to use MD PBS at follow-up. Participants’ age and gender, alcohol use measures (frequency of alcohol, drunkenness, and quantity of alcohol consumed), and intention to use MD PBS at baseline were included as control variables in all the regression models.

Mplus 8.7 ( Muthén and Muthén 1998-2017) multigroup regression analyses were used to evaluate the invariance of the models across gender. Multigroup models were specified with all the regression coefficients restricted to be equal for men and women. Three indices were used to evaluate the fit of the model: root mean square error of approximation (RMSEA), comparative fit index (CFI), and standardized root mean squared residual (SRMR). Accepted CFI values are >0.95, and the RMSEA and SRMR should be <0.08 (Hu and Bentler 1999). The fit of the constrained models was considered a measure of the plausibility of the invariance hypotheses.

## Results

### Descriptive and bivariate results

Table 1 presents means, standard deviations, and bivariate correlations between predictors and outcome variables. The baseline perceived severity score was positively related to the intention to use all behaviors except “Avoiding drinking games” and “Avoiding pregaming”. In contrast, the baseline perceived vulnerability score was not associated with the intention to use any of the behaviors. Regarding coping components, perceived efficacy and self-efficacy at baseline were positively associated with the intention to use PBS at follow-up.

**Table 1:** Descriptive statistics and correlations between study variables.

		Intention ADG	Intention AM	Intention DS	Intention AKU	Intention AP	Intention TM
	*M* (SD)	11.77 (5.90)	15.84 (5.18)	15.27 (4.96)	16.04 (4.71)	12.39 (5.45)	71.60 (20.46)
Age	21.15 (2.23)	0.144	0.145	0.154	0.129	0.129	0.188^*^
Alcohol use	15.49 (11.39)	−0.206^*^	−0.254^*^	−0.295^*^	−0.201^*^	−0.285^*^	−0.301^*^
Drunkenness	5.70 (7.11)	−0.277^*^	−0.301^*^	−0.393^*^	−0.284^*^	−0.321^*^	−0.393^*^
Alcohol use quantity (SDU)	20.61 (18.20)	−0.067	−0.037	−0.016	−0.012	−0.044	−0.045
Perceived severity	8.25 (1.35)	0.122	0.171^*^	0.252^*^	0.236^*^	0.067	0.202^*^
Perceived vulnerability	6.29 (1.98)	0.042	0.013	−0.047	−0.009	−0.022	−0.021
Perceived efficacy of ADG	3.02 (0.88)	0.306^*^	0.156	0.225^*^	0.171^*^	0.219^*^	0.282^*^
Perceived efficacy of AM	3.60 (0.70)	0.101	0.307^*^	0.241^*^	0.196^*^	0.091	0.241^*^
Perceived efficacy of DS	3.54 (0.67)	0.108	0.229^*^	0.315^*^	0.281^*^	0.171^*^	0.282^*^
Perceived efficacy of AKU	3.39 (0.82)	0.115	0.164^*^	0.233^*^	0.252^*^	0.102	0.213^*^
Perceived efficacy of AP	2.87 (0.87)	0.122	0.181^*^	0.115	0.079	0.294^*^	0.202^*^
Perceived efficacy of TM	16.43 (2.60)	0.240^*^	0.305^*^	0.330^*^	0.293^*^	0.277^*^	0.371^*^
Perceived self-efficacy of ADG	18.74 (3.32)	0.185^*^	0.187^*^	0.241^*^	0.221^*^	0.104	0.245^*^
Perceived self-efficacy of AM	19.32 (2.53)	0.062	0.227^*^	0.234^*^	0.243^*^	0.073	0.212^*^
Perceived self-efficacy of DS	18.64 (3.34)	0.107	0.205^*^	0.407^*^	0.302^*^	0.134	0.303^*^
Perceived self-efficacy of AKU	18.86 (2.98)	0.086	0.211^*^	0.304^*^	0.333^*^	0.133	0.274^*^
Perceived self-efficacy of AP	18.86 (3.59)	0.088	0.076	0.193^*^	0.211^*^	0.232^*^	0.205^*^
Perceived self-efficacy of TM	94.39 (11.39)	0.150	0.251^*^	0.387^*^	0.375^*^	0.197^*^	0.351^*^

*Note*. ADG: avoiding drinking games; AM: avoiding mixing different types of alcohol; DS: drinking slowly; AKU: avoiding keeping up; AP: avoiding pregaming; TM: total score in the MD subscale; SDU: standard drink units.

Bonferroni adjustment ^*^P < .003.

### Regression results

The results of the multiple regression predicting intention to use MD PBS as a function of the PMT variables are shown in Table 2. After controlling for the effects of sociodemographic variables, alcohol use, and intention to use MD PBS at baseline, the variance explained by the PMT constructs ranged from 7.5% for the intention to avoid drinking games to 16% for the intention to drink slowly. Perceived efficacy and perceived self-efficacy were identified as significant predictors of the intention outcome in all the regression models (Table 2). On the other hand, while perceived vulnerability was not a significant predictor of any intention outcomes, perceived severity was associated with the intention to use three out of five MD PBS. All the significant coefficients indicated a positive association between the PMT predictors and the intentions to engage in MD strategies.

**Table 2:** Hierarchical multiple regression examining the association between PMT variables at baseline and the intentions to use MD PBS at follow-up, controlling for sociodemographic variables, alcohol use measures, and intention of the use MD PBS.

**Outcomes and predictor**	***B***	**95% CI**	***β***	***P***	***Sr*** ^***2***^	***ΔR*** ^***2***^
**Intention to avoid drinking games at follow-up**						
Gender	−1.01	[−2.26, 0.24]	−0.09	0.113	0.006	
Age	0.36	[0.07, 0.64]	0.13	0.013	0.017	
Frequency of alcohol use at baseline	−0.01	[−0.09, 0.06]	−0.03	0.717	0.000	
Frequency of drunkenness at baseline	−0.14	[−0.25, −0.03]	−0.17	0.016	0.016	
Alcohol use quantity at baseline	−0.02	[−0.05, 0.02]	−0.06	0.282	0.003	
Intention to avoid drinking games at baseline	−0.02	[−0.14, 0.09]	−0.02	0.712	0.000	0.100^*^^*^^*^
Perceived vulnerability at baseline	0.01	[−0.32, 0.35]	0.00	0.939	0.000	
Perceived severity at baseline	0.16	[−0.33, 0.65]	0.04	0.520	0.001	
Perceived efficacy at baseline	**1.53**	**[0.79, 2.28]**	**0.23**	**<0.001**	**0.045**	
Perceived self-efficacy at baseline	**0.22**	**[0.03, 0.41]**	**0.12**	**0.027**	0.**014**	0.075^***^
**Intention to avoid mixing at follow-up**						
Gender	0.23	[−0.86, 1.32]	0.02	0.678	0.000	
Age	0.22	[−0.03, 0.48]	0.09	0.082	0.008	
Frequency of alcohol use at baseline	−0.03	[−0.09, 0.04]	−0.05	0.477	0.001	
Frequency of drunkenness at baseline	−0.15	[−0.25, −0.05]	−0.20	0.003	0.024	
Alcohol use quantity at baseline	−0.01	[0.04, 0.02]	−0.02	0.693	0.000	
Intention to avoid mixing at baseline	−0.03	[−0.14, 0.08]	−0.03	0.600	0.000	0.118^*^^*^^*^
Perceived vulnerability at baseline	−0.13	[−0.41, 0.16]	−0.05	0.391	0.001	
Perceived severity at baseline	**0.55**	**[0.14, 0.96]**	**0.14**	**0.010**	**0.018**	
Perceived efficacy at baseline	**1.61**	**[0.83, 2.40]**	**0.22**	**<0.001**	**0.043**	
Perceived self-efficacy at baseline	**0.25**	**[0.03, 0.48]**	**0.12**	**0.028**	**0.013**	0.087^*^^*^
**Intention to drink slowly at follow-up**						
Gender	0.50	[−0.43, 1.42]	0.05	0.295	0.002	
Age	0.27	[0.05, 0.49]	0.12	0.015	0.013	
Frequency of alcohol use at baseline	0.01	[−0.05, 0.06]	0.02	0.803	0.000	
Frequency of drunkenness at baseline	−0.20	[−0.28, −0.12]	−0.29	<0.001	0.050	
Alcohol use quantity at baseline	−0.01	[−0.03, 0.02]	−0.02	0.610	0.000	
Intention to avoid drink slowly at baseline	0.03	[−0.07, 0.13]	0.03	0.609	0.000	0.179^*^^*^^*^
Perceived vulnerability at baseline	−0.20	[−0.45, 0.05]	−0.08	0.119	0.005	
Perceived severity at baseline	**0.63**	**[0.26, 0.99]**	**0.17**	**0.001**	**0.025**	
Perceived efficacy at baseline	**1.09**	**[0.32, 1.86]**	**0.14**	**0.005**	**0.017**	
Perceived self-efficacy at baseline	**0.41**	**[0.25, 0.56]**	**0.27**	**<0.001**	**0.060**	0.155^*^^*^
**Intention to avoid keeping up at follow-up**						
Gender	0.71	[−0.25, 1.67]	0.08	0.144	0.005	
Age	0.21	[−0.01, 0.43]	0.10	0.066	0.008	
Frequency of alcohol use at baseline	−0.00	[−0.06, 0.05]	−0.01	0.932	0.000	
Frequency of drunkenness at baseline	−0.11	[−0.20, −0.03]	−0.18	0.009	0.017	
Alcohol use quantity at baseline	0.00	[−0.03, 0.03]	0.01	0.912	0.000	
Intention to avoid keeping up at baseline	0.02	[−0.08, 0.13]	0.02	0.695	0.000	0.102^*^^*^^*^
Perceived vulnerability at baseline	−0.20	[−0.45, 0.06]	−0.08	0.140	0.005	
Perceived severity at baseline	**0.64**	**[0.27, 1.01]**	**0.19**	**0.001**	**0.030**	
Perceived efficacy at baseline	**0.97**	**[0.36, 1.57]**	**0.17**	**0.002**	**0.025**	
Perceived self-efficacy at baseline	**0.32**	**[0.15, 0.50]**	**0.20**	**<0.001**	**0.034**	0.123^*^^*^
**Intention to avoid pregaming at follow-up**						
Gender	−0.22	[−1.32, 0.89]	−0.02	0.699	0.001	
Age	0.25	[−0.01, 0.51]	0.10	0.057	0.009	
Frequency of alcohol use at baseline	−0.05	[−0.11, 0.02]	−0.10	0.142	0.005	
Frequency of drunkenness at baseline	−0.13	[−0.24, −0.03]	−0.18	0.009	0.018	
Alcohol use quantity at baseline	−0.00	[−0.03, 0.03]	−0.01	0.930	0.000	
Intention to avoid pregaming at baseline	0.03	[−0.07, 0.14]	0.03	0.555	0.000	0.130^*^^*^^*^
Perceived vulnerability at baseline	−0.08	[−0.38, 0.22]	−0.03	0.603	0.000	
Perceived severity at baseline	−0.04	[−0.47, 0.39]	−0.01	0.856	0.000	
Perceived efficacy at baseline	**1.65**	**[1.01, 2.29]**	**0.26**	**<0.001**	**0.067**	
Perceived self-efficacy at baseline	**0.24**	**[0.07, 0.41]**	**0.15**	**0.005**	**0.021**	0.090^*^^*^
**Intention, total score in** MD **at follow-up**						
Gender	−0.02	[−4.09, 4.06]	0.01	0.994	0.000	
Age	1.33	[0.39, 2.27]	0.14	0.006	0.018	
Frequency of alcohol use at baseline	0.03	[−0.21, 0.26]	0.02	0.816	0.000	
Frequency of drunkenness at baseline	−0.75	[−1.11, −0.38]	−0.26	<0.001	0.040	
Alcohol use quantity at baseline	−0.00	[−0.12, 0.11]	−0.00	0.946	0.000	
Intention, total score in MD at baseline	0.05	[−0.06, 0.17]	0.05	0.357	0.005	0.190^*^^*^^*^
Perceived vulnerability at baseline	−0.84	[−1.94, 0.27]	−0.08	0.137	0.005	
Perceived severity at baseline	1.50	[−0.09, 3.07]	0.10	0.064	0.008	
Perceived efficacy at baseline	**1.98**	**[1.08, 2.88]**	**0.24**	**<0.001**	**0.045**	
Perceived self-efficacy at baseline	**0.34**	**[0.14, 0.55]**	**0.18**	**0.001**	**0.025**	0.125^*^^*^^*^

*Note*: Hierarchical regression steps: Step 1 included sociodemographic variables, alcohol use measures, and intention to use each MD PBS at baseline; Step 2 added PMT variables. The parameters of the final model are presented, and the significant variables of the PMT are indicated in bold type. PBS = protective behavioral strategies.

^**^ *P* < 0.01, ^***^P < 0.001.1

The fit of the six multigroup gender-invariant models is presented in Table 3. None of the model chi-squares were significant, indicating that regression coefficients were gender invariant for the six models.

**Table 3:** Multigroup gender-invariant regression models.

	Chi-square *(df)*	*P*	RMSEA	CFI	SRMR
Avoiding drinking games	2.39 (8)	0.967	0	1	0.023
Avoiding mixing	2.52 (8)	0.961	0	1	0.025
Drinking slowly	6.28 (8)	0.616	0	1	0.036
Avoiding keeping-up	7.75 (8)	0.458	0	1	0.040
Avoiding pregaming	6.70 (8)	0.569	0	1	0.043
Total MD score	5.54 (8)	0.699	0	1	0.035

*df*: degrees of freedom; *P*. *P* value; RMSEA: root mean-square error of approximation; CFI: comparative fit index; SRMR: standardized root mean-square residual; MD: manner of drinking.

## Discussion

Theory-based interventions have demonstrated utility in promoting various health-related behaviors (e.g. DiClemente et al. 2013). However, despite research examining the psychological determinants of alcohol PBS use (González-Ponce et al. 2022b), theory-based research is scarce in this area. To our knowledge, this is the first study to prospectively examine the explanatory value of one of the most prominent theories in the field of health-related behavior, the PMT (Maddux and Rogers 1983), for the intention to use alcohol MD PBS among young adults. Our findings support the utility of PMT when applied to the intention to use MD PBS and indicate that its explanatory value does not differ according to gender.

As hypothesized and consistent with previous studies and systematic reviews (e.g. Ruiter et al. 2014), our findings support the greater explanatory value of coping appraisal (response efficacy and self-efficacy) compared to threat appraisal (vulnerability and severity). However, the variance explained by these models was limited. These findings align with previous studies showing an association between coping appraisal components (response efficacy and self-efficacy) and PBS use ( González-Ponce et al. 2022a). Moreover, our findings support the assertion made by Ruiter et al. (2014) following their meta-analytic review, who highlighted that presenting threatening health-related information (i.e. fear appeal) has a limited impact on promoting protective behaviors. In contrast, promoting MD PBS efficacy to reduce alcohol-related negative consequences and increasing perceived self-efficacy to use MD PBS appear to be more effective ways of increasing the motivation to use alcohol MD PBS among young adults.

Regarding the threat appraisal components, previous meta-analytic reviews have demonstrated the greater utility of perceived severity in explaining health-related behaviors (e.g. Bui et al. 2013), while others highlight perceived vulnerability as the most explanatory component of threat appraisal (e.g. Milne et al. 2000). Our results suggest the importance of perceived severity since no prospective association was found between perceived vulnerability and MD PBS intentions. However, perceived severity was associated with the intention to use three out of five PBS and with the total MD PBS score, which suggests that providing information about the severity of alcohol’s negative consequences may encourage alcohol-using young adults to use MD PBS.

In line with previous research (e.g. Ruiter et al. 2014), beliefs about response efficacy and the ability to engage in MD PBS may be the best predictors of intentions to use MD PBS. Previous meta-analytic reviews (e.g. Bui et al. 2013) have indicated that self-efficacy is a stronger predictor than response efficacy. These findings are consistent with the significance of self-efficacy as a core construct in other prominent theories in the field of human behavior, such as the theory of planned behavior (Ajzen 1991) and social cognitive theory (Bandura 1977). However, our findings do not fully support the superiority of self-efficacy over response efficacy since the association between baseline perceived self-efficacy and follow-up intention was stronger than that between response efficacy and intention for only two out of the five MD PBS (“Drinking slowly” and “Avoiding keeping up or outdrinking others”). Thus, both coping components seem to have a comparable impact on the intention to use alcohol PBS.

As consistently shown in previous research (e.g. Schwebel et al. 2022), women tend to use more PBS than men. This difference may be attributed to women’s higher affective response to health threats compared to men. However, our results indicate that the relationships between PMT constructs and intentions to use the five alcohol MD PBS examined were invariant across genders. This finding is in line with the results reported by Plotnikoff et al. (2009), who found gender invariance when examining the explanatory value of the PMT in the field of physical activity. These results suggest that PMT-based interventions could similarly impact both men and women.

### Practical implications

The results of this study offer recommendations within the PMT framework for promoting intentions to use MD PBS. While previous studies have highlighted the utility of fear appeals in promoting intentions to use protective behaviors (Stainback and Rogers 1983), our findings suggest that promoting threat perception (e.g. “Drinking too much alcohol too quickly can lead to alcohol poisoning, which can kill you”; Cismaru et al. 2008; p. 285) will have a limited impact on protection motivation.

In contrast, our results reveal that strengthening young adults’ protection motivation could involve fostering self-efficacy and perceived efficacy of alcohol MD PBS (i.e. positive expectations of PBS; González-Ponce et al. 2022a). This could be achieved, for instance, through vicarious experience, demonstrating to young adults that their peers successfully use MD PBS to reduce the negative consequences of alcohol (i.e. social comparison processes; Bandura 1977). Additionally, these interventions could address the negative expectancies of PBS use, that is, the possibility that such use might diminish the positive effects sought from drinking alcohol. For example, providing information such as the following could enhance perceived self-efficacy and the effectiveness of MD PBS: “Most young people use protective strategies when they drink alcohol. Research indicates that individuals aged 18-25 who utilize these strategies achieve the desired outcome of a fun and successful party without suffering negative consequences.”

### Limitations and future directions

Several limitations should be considered when interpreting our results. First, previous research (e.g. Witte and Allen et al. 2000) suggests that fear appeal may be more effective when threats are explicitly described using specific examples of particular negative consequences (e.g. loss of consciousness, vomiting) rather than referring to a broad category of negative consequences. However, in our study, while we asked about the perceived vulnerability and severity of health consequences of drinking and binge drinking, we did not provide concrete examples of such threats. This aspect of our procedure might explain why we observed a lower explanatory power of threat appraisal than coping appraisal.

Additionally, the internal consistency reliability was only acceptable for the perceived efficacy of the MD scale (*α* = *0*.77), which may have influenced the explanatory power of this construct in our research. Moreover, it should be noted that our study did not use a probabilistic sampling procedure, limiting our ability to generalize our findings to the wider alcohol-using young adult population. Furthermore, since our study focused solely on MD PBS, the generalizability of our findings to other protective behaviors (i.e. SHR or SLD) is questionable. Additionally, as this study did not examine the explanatory value of the PMT for other protective strategies, future research should address this issue, particularly concerning SHR PBS, which have shown notable effectiveness in reducing alcohol-related consequences. Moreover, using a single 2-month follow-up may have affected our ability to accurately predict the intention to use MD PBS. Future studies should, therefore, consider different time periods (i.e. longer or shorter) to obtain a more precise understanding of the predictive capacity of PMT.

It is important to note that while our study examined the explanatory value of PMT for motivation (i.e. intention) to use alcohol PBS, we did not include a second follow-up survey to collect information about actual PBS use. Given that behavioral intention only partially explains future behavior—the intention–behavior gap (Conner and Norman 2022), future studies should include a follow-up measure of behavior to determine the extent to which the intention to use PBS translates into the actual use of these strategies in the short term. Additionally, it would be of interest to include representative samples of young adults and more specific measures of potential alcohol-related consequences in future studies.

Finally, our study used the original version of the PMT by Maddux and Rogers (1983), which may explain the limited variance explained by this model. Therefore, future studies should explore the explanatory power of the extended model (Rogers 1983), incorporating the rewards (intrinsic and extrinsic) of risk behavior and the costs of protection behavior.

## Conclusions

Our findings demonstrate the utility of the PMT in explaining the intention to use a set of alcohol MD PBS, which are associated with less intensive alcohol consumption and fewer negative consequences. In particular, our findings suggest that interventions aimed at promoting the intention to use alcohol MD PBS among young adults could particularly benefit from including components of the PMT, encouraging self-efficacy to use MD PBS and emphasizing the perceived effectiveness of such strategies.

## Data Availability

The data supporting this article will be shared upon reasonable request to the corresponding author (F.F.C.).

## Open access

Funding for open access charge: Universidad de Huelva / CBUA.
